# Introduction: mapping the multiculturalism-interculturalism debate

**DOI:** 10.1186/s40878-018-0080-8

**Published:** 2018-05-17

**Authors:** François Levrau, Patrick Loobuyck

**Affiliations:** Centre Pieter Gillis, University of Antwerp, Prinsstraat 13, 2000 Antwerp, Belgium

**Keywords:** Multiculturalism, Interculturalism, Backlash against multiculturalism, Integration, Ethnocultural minorities

## Abstract

Since the 1970s multiculturalist policies that recognize and accommodate ethnocultural diversity have been implemented across western democracies. However, the tide seems to have changed: a ‘backlash against multiculturalism’ has been occurring since the 1990s. While it remains unclear whether this backlash is a matter of rhetoric or if there is indeed a wholesale retreat from multiculturalism, several scholars, politicians and journalists have invoked a pervasive narrative of the rise/advance and fall/retreat of multiculturalism. ‘Interculturalism’ has been introduced as a remedy, being allegedly well-suited to address some of the shortcomings of the multicultural approach. In this introduction to the Special Issue, which is about the key texts of Tariq Modood and Ricard Zapata Barrero, we present and question the nexus between the two terms. How has the ‘multiculturalism-interculturalism’ debate been held so far?

## Introduction

From the 1960s-1970s onwards, multiculturalism has had both its proponents and critics. While the support consistently outweighed the critique for the first decades, the tide seemed to change at the beginning of the new millennium. As critical voices have become more numerous and vigorous, most theorists and politicians would be rather reluctant to assert what Will Kymlicka (2001) victoriously stated some 20 years ago, namely that multiculturalists have won the day in defending difference-conscious notions of justice and concomitant laws and policies. Moreover, the critiques have increasingly merged into a growing chorus of concern and a popular refrain about the failure, decline and even death of multiculturalism as a political philosophy as well as a particular type of policy. Much of the animosity towards multiculturalism, however, involves immigrant-driven diversity, in particular Muslim immigrants, as there is no similar retreat from the commitment to multicultural citizenship for indigenous peoples or national minorities (Kymlicka, 2012).^1^ In fact, multiculturalism is criticized for numerous reasons (Vertovec & Wessendorf, 2010b). It allegedly involves cultural relativism, attributes to culture an unchanging essence, treats culture as a determinant of human behaviour, encourages segregation and eventually leads to social fragmentation. Multiculturalism is supposedly also obsessed with cultural difference, thereby disregarding the importance of common values. And, if things were not bad enough already, multiculturalism is apparently also bad for women and animals, and additionally, some have even accused the multicultural paradigm for leading to terrorism.

The critiques that have been formulated so far are twofold, as the charge that multiculturalism is tantamount to “unfair privileges or invidious forms of discrimination” has been increasingly complemented by the assertion that multiculturalism can erode civic virtues, identities and practices that are necessary for a healthy democracy. In that regard Kymlicka (2001) points at a “second front in the ‘multiculturalism wars’”: critics are increasingly rejecting the idea of minority rights on the basis of some perverse social effects (see e.g. Koopmans, 2010). One of the most influential critiques was launched by Brian Barry (2001), who accused multiculturalism of being anti-universalistic and anti-liberal, thereby denying the ideals of the Enlightenment. Barry also argued, however, that multiculturalism unjustly supports the politicization of cultural group identities, thereby paradoxically obstructing the integration of minorities. As such, he was an important spokesman for the critique that multiculturalism can undermine social cohesion, the necessary social condition for socio-economic redistribution. *‘A politics of multiculturalism undermines a politics of redistribution’* (Barry, 2001, pp. 8, 321). The more a policy offers public recognition, accommodation, support and status to ethnocultural differences, the less attention it pays to social unity, the very foundation upon which a policy of redistribution is based. This criticism has been called the ‘recognition-redistribution trade-off’ (Banting & Kymlicka, 2006) as it suggests a trade-off between the recognition of diversity on the one hand and the maintenance of social unity and socioeconomic redistribution on the other. This also bears similarity with the so-called Putnam thesis or the ‘heterogeneity-redistribution trade-off’ (Banting & Kymlicka, 2006). According to Robert Putnam (2007) there is a negative relation between the presence of ethnic diversity and indicators of social cohesion such as mutual trust and solidarity. Putnam (2007, p. 151) famously argued that ‘*[d]iversity, at least in the short run, seems to bring out the turtle in all of us.’* Pearce (2004) has described this as a ‘progressive dilemma’. The argument runs like this: *‘(...) the more different we become from one another – the more diverse our ways of life and our religious and ethnic backgrounds – and the less we share a moral consensus or a sense of fellow feeling, the less happy we will be in the long run to support a generous welfare state’* (Goodhart, 2006, p. 16). The relation between heterogeneity and solidarity has been the subject of many studies – not in the least because Putnam believed to have found a ‘social law’ and therefore invited fellow researchers from all over the world to examine his thesis. The broad post-Putnam research, in fact, has led to what Van der Meer & Tolsma (2014) call a ‘cacophony of empirical findings’. Based on cross-national data from 28 European countries, Gesthuizen, Van der Meer and Scheepers (2009, p. 121) conclude that *‘Putnam’s hypothesis on ethnic diversity must be refuted in European societies.’* Uslaner (2012), for example, showed that it is not diversity as such, but rather the degree of social isolation and (residential) segregation that leads to a decrease in mutual trust and social cohesion.

In a time where ‘multiculturalism’ has become the proverbial punching bag, ‘interculturalism’ has been increasingly put forward as a new, distinct and very welcome alternative. Interculturalism gives special attention to social interaction, contacts between people of different backgrounds and shared membership. Whether or not interculturalism indeed provides a fundamentally new paradigm, the idea of interpersonal contact as a tool to create a stronger sense of belonging together might be an important way to counter Barry’s criticism (see Levrau & Loobuyck, 2013a, 2013b). After all, it could be argued that solidarity has continued to exist in current welfare states precisely because they have not only implemented multiculturalism and liberal nationalism on the macro level, but have also invested in interculturalism at the meso and micro level. So, regardless of whether the idea of interpersonal relations and a shared sense of belonging together is already to be found in the multicultural frame, interculturalism highlights an important element. After all, the importance of ‘interpersonal contact’ cannot be emphasized enough, given today’s socio-political context of superdiversity, nationalism and globalization. This is why we very much appreciate that *Comparative Migration Studies* has given us the opportunity to elaborate these ideas and to further discuss the nexus between multiculturalism and interculturalism.

### The ever growing multicultural library

Multiculturalism is both a political philosophical paradigm and a type of policy that is particularly sensitive to the possibility that liberal democracies could exert pressure on ethnic minorities to assimilate into the majority culture. Multiculturalism – irrespective of the many variances it contains – defends the idea that the societal institutions need to provide the same degree of respect, recognition and accommodation to the identities and practices of ethnocultural minority groups as they traditionally have to the identities and practices of the majority group. In order to prevent the obligation or expectation that minorities speak the language of the majority, or adopt its customs and lose their distinctiveness, multiculturalists favour all sorts of minority measures or group rights to protect and/or promote linguistic, ethno-cultural and religious diversity. This paradigm has been influential since the 1970s – Kymlicka (2007) has argued that multiculturalism is to be considered as a part of a larger human rights revolution involving ethnic and racial diversity that occurred after WWII – and has given rise to an ever expanding academic ‘multicultural library’.

Kymlicka (2002) has illustrated how the debate on the rights of ethnocultural minorities took three steps. In the 1970s and 1980s the debate was essentially framed in terms of the old controversy between liberals and communitarians. The debate was revitalized by John Rawls (1971) as it gave rise to fierce critiques from so-called communitarians like Michael Sandel (1982), Michael Walzer (1983) and Charles Taylor (1992). At that time, the discussion basically came down to a disagreement about the way people stand in the world: either as individuals (position of liberalism) or as members of a group (position of communitarianism). Multiculturalists of the first hour supported their plea largely by referring to the communitarian framework. In the second phase, claims for recognition and support for ethnocultural minority groups are defended within the liberal framework. Kymlicka (1989) was the one who gave the proverbial starting shot of this second phase by arguing that a (societal) culture provides a context of choice which is necessary for the individual autonomy to flourish. In a third phase, multicultural rights are defended as compensations for the nation building of the majority group. As such, *“The question is no longer how to justify departure from a norm of benign neglect, but rather, do majority efforts at nation-building create injustices for minorities? And if so, do minority rights help protect against these injustices”* (Kymlicka, 2002, p. 347)?

Over the years an enormous number of books have been published on the rights of ethnocultural minorities. As such the literature on whether or not individuals can expect to be recognized for their membership to a cultural group has become vast and to a certain degree labyrinthine. Within the many nuances, however, three broad and distinctive trends can be identified – although we are sure that many other taxonomies can be made.^2^

A first group of authors asserts that the claims of cultural minorities should not be accommodated by means of group rights. Barry (2001), for example, rejects special cultural rights on the basis of equal treatment and a concern for state-sponsored social justice. Liberal egalitarianism cannot tolerate multiculturalism because it would condemn liberal rights, lead to societal fragmentation and take away the attention from socioeconomic inequalities. For Moller Okin (1998) multicultural recognition is basically a way to protect and preserve traditions that are fundamentally patriarchal. There is a trade-off: either one supports a multiculturalist commitment to group rights for ethnic-cultural minorities and thus is willing to pay the price of gender equality, or one defends ‘feminism’ and rejects multiculturalism as it would be ‘bad for women’. Kukathas (2003) advocates minimal state intervention and maximal negative liberty for individual citizens. According to Kukathas liberalism is not concerned with granting recognition as, in his view, liberalism is already a theory of multiculturalism, pluralism and diversity. His theory is based on the idea of tolerance and freedom of association, leading to a society that can be described as a kind of archipelago of communities. While their respective theories differ in a lot of aspects and while these authors have criticized each other’s work, it is remarkable that they believe that a commitment to their liberal paradigm implies that one should lose the element of group rights. Phillips (2007) and Joppke (2017) have also been rather critical towards the idea of multicultural group rights. Both authors advocate a simple humanism that stresses the basic similarities in the ways people behave and a form of multiculturalism grounded in the rights of the individual rather than those of groups.

A second group of authors advocates communitarian group rights by emphasizing cultural groups’ moral authority as entities in themselves, or by referring to cultures as significant identity-conferring associations. Both the seminal work of Taylor (1992) and the influential work of Parekh (2000b) might be exemplary. Both Taylor and Parekh have not built their theories upon the assumption that a culture *“helps individuals develop their capacity for autonomy, which then transcends it and views it and the wider world untainted by its provenance”* (Parekh, 2000b, p.110). According to Taylor and Parekh there are no such uprooted or transcendent beings and therefore culture as such needs a kind of protection. Taylor (1992), for example, argues that one must respect the different cultures as they have actually evolved. Moreover, these cultures need to survive and their value needs to be acknowledged. In his much cited essay *‘The politics of recognition’* he upholds that citizens of Quebec should be subject to restrictions imposed by their government. While these restrictions would be annulled in other parts of Canada, Taylor defends them in the name of the collective purpose of the survival of the culture of Quebec.

A third group does not wish to implement group rights as a means of representing a group as such (because the group or culture has a value in its own), but as a means of enhancing the freedom and equality of individuals in a shared societal context. Here, we might think of Kymlicka (1989, 1995), but also of Raz (1994). Kymlicka has proposed an influential liberal cultural theory in which it is argued that certain types of multicultural rights are consistent with liberal democratic principles and that common objections on the grounds of individual freedom, social justice and national unity should not have the last word, as they can be reasonably solved. A recent attempt to advance the debate beyond the well-known multicultural philosophers is from Patten (2014), who has made a strong case for cultural rights as requirements of liberal justice. His justification of minority rights is based on a strong appeal to the ideals of philosophical liberalism, such as state neutrality, individual self-determination, and pluralism about conceptions of the good life.

While these three theoretical perspectives differ greatly, they share the assumption that the discussion is about how a neutral government should treat the ethnocultural (and associated religious) identities of its citizens. While much attention has been given to the recognition of difference, in the oeuvre of scholars like Modood (2007) it is a recurrent theme that the recognition of difference by no means precludes attention for a shared sense of belonging together. Kymlicka (2001) as well has linked his multiculturalism project to a liberal nationalism position in order to emphasize that multiculturalism does not necessarily lead to a compilation of *Parallel-Gemeinschaften*. He rejects both the traditional liberal view that shared beliefs in universal principles of justice are sufficient for a sustainable practice of democracy and social justice (the benign neglect), and the communitarian view that shared beliefs in a particular conception of the good – and thus a non-neutral politics of the common good – are necessary for the practice of democracy and social justice. Citizens need to share more than simple political principles, but less than a shared (communitarian) conception of the good life. A shared, but ‘thin’ national identity should and can be sufficient.

### Multiculturalism in practice

The valuing of ethnocultural diversity in balance with equality of opportunity and mutual tolerance has led to multicultural policies, first in Canada and Australia in the early 1970’s, and in many other liberal democracies soon after. Banting & Kymlicka (2006, pp. 56-57) have listed the following concrete multicultural policies that have been implemented for ethnocultural immigrant groups.^3^

1. Constitutional, legislative or parliamentary affirmation of multiculturalism at the central and/or regional and municipal levels and the existence of a government ministry, secretariat or advisory board to implement this policy in consultation with ethnic communities.
2. Adoption of multiculturalism in school curricula.
3. Inclusion of ethnic representation/sensitivity in the mandate of public media or media licensing.
4. Exemptions from dress codes (either by statute or court cases).
5. Dual citizenship arrangements.
6. Funding of bilingual education or mother-tongue instruction.
7. Affirmative action for disadvantaged immigrant groups.

Curiously, and in contrast to what the ‘multiculturalism is dead’ rhetoric suggests, these policies have been increasingly implemented in many Western countries (Kymlicka, 2012). Moreover, some of the countries proclaiming the idea of a ‘failed multiculturalism’ had never implemented multicultural policies in the first place. Some scholars have therefore argued that the open hostility to multiculturalism is [for some] an exercise in avoiding the term ‘multiculturalism’ rather than a real retreat from its principles (McGhee, 2008). Vertovec & Wessendorf (2010a, p. 18) contend that even if the *word* multiculturalism may have increasingly disappeared from political rhetoric, this has not led to the *‘eradication, or even the detriment, of actual measures, institutions, and frameworks of minority cultural recognition’.* If multiculturalist policies are indeed still being implemented in most Western countries, it has been alongside new civic integration policies which (1) focus on the role of employment in integration; (2) expect newcomers to respect basic liberal-democratic values; and (3) emphasize basic knowledge of the host society’s language, history and institutions (Kymlicka, 2012). While Joppke (2004) argues that the emergence of these policies is an indication of how under threat multiculturalism is, Kymlicka maintains that both policy types can be implemented simultaneously. According to Levrau & Loobuyck (2013b); see also Loobuyck (2016) it is even possible to consider civic integration policies as a part of multiculturalism and interculturalism.

As mentioned, the first multicultural policies emerged in the late 1960s and early 1970s in countries like Canada and Australia, and to a lesser extent in the US and UK. In the *US*, multiculturalism is closely linked to the Civil Rights Movements and thus to a ‘bottom up’ protest movement whereby African-Americans and other identity groups fought against racial segregation and discrimination and strived for legal recognition and federal protection of the citizenship rights enumerated in the Constitution and federal law. Accordingly, multiculturalism in the US is strongly associated with affirmative action programmes and with ‘multicultural school policies and curricula’. In the *UK*, multiculturalism emphasized the importance of curricula that take sufficient account of cultural diversity and the importance of education in the native language and culture of immigrants. ‘Multiculturalism’ was the driving force behind the Swann Report on multicultural education in Britain in 1985. Scholars like Stuart Hall, Tariq Modood and Bhikhu Parekh further emphasized the need to rethink the national story so that all people are/feel included. This was, in fact, the most important message of the famous ‘Parekh Report’ (Parekh, 2000a). In *Canada*, on the other hand, multiculturalism had a link with discussions about the constitution and territory. Multiculturalism defended the separate status and separate interests and rights of indigenous peoples and nationalist, separatist minority groups (i.e. French-speaking Québec). From the beginning, multiculturalism in Canada was explicitly proposed as a liberal policy, which would thus ratify individual freedoms and equality and the importance of equal citizenship through the implementation of group rights. For example, Canadian Prime Minister Pierre Elliot Trudeau stated that Canada would pursue a multicultural policy in which diversity at the level of language, culture and religion would be recognized and respected in order to increase equality and freedom among the Canadian citizens. In 1982, this was formally recognized in section 27 of the Canadian Charter of Rights and Freedoms, and in 1988 the Multiculturalism Act was implemented, making the first multicultural laws in Canada a reality. In *Australia,* multiculturalism was implemented as an alternative to the assimilation policy that expected rapid adaptation from immigrants (Levey, 2001). The policy did not include Indigenous Australians until the end of the 1970s and the Galbally Report of 1978 which spoke of multiculturalism being a policy for all Australians including Indigenous Australians. This inclusive approach was first formally included in the National Agenda for a Multicultural Australia, and became the first multicultural policy implemented under the Hawke Labour government in 1989.

Multiculturalism differs depending on the type of multicultural diversity and the type of claims that a state faces. Multiculturalism in countries such as Canada (and Australia) is now mainly associated with indigenous peoples and minorities that wish to achieve a form of autonomy. Many debates about multicultural society in North America are about whether these groups are entitled to self-government. In other Western countries, especially in Europe (e.g. *UK and the Netherlands*), multiculturalism is primarily associated with immigrant and colonial minority policies. The multicultural debate in Western Europe is highly determined by the question whether and to what extent integration can be combined with recognition of certain elements of the ethnic-cultural identity of immigrants. “*(…) the term “multiculturalism” in Europe came to mean, and now means throughout the English-speaking world and beyond, the political accommodation by the state and/or a dominant group of all minority cultures defined first and foremost by reference to race, ethnicity or religion, and, additionally but more controversially, by reference to other group-defining characteristics such as nationality and aboriginality”* (Meer & Modood, 2016, p.34). The ‘multiculturalism debate’, however, has been increasingly dominated by what Parekh (Parekh, 2008; see also Modood, Triandafyllidou, & Zapata-Barrero, 2006) has coined the ‘Muslim question’. The ‘headscarf debates’ in particular are exemplary for how the Western world is ‘struggling’ with Islam. Joppke (2009) famously argued that while each country had the headscarf controversy it deserved, there simply is no country in the Western world – despite highly divergent legacies of state, religion and nationhood – that has not been affected by a ‘headscarf debate’.

### Interculturalism: A new paradigm?

Given the diversity of (often contrasting) voices, it is fair to say that there is simply no such thing as ‘a multicultural theory’ that then could be affirmed, rejected or revised as a whole. There is rather a ‘multicultural discipline’ that speaks to a lot of scholars who are working, most prominently, in the field of political philosophy and political studies, but also in educational science, sociology, cultural studies, anthropology, etc. Since the existence of this complexity and huge number of (contrasting) voices, ‘interculturalists’ could be depicted as a relatively recent group of authors who have injected their ideas in what we have called ‘the ever growing multicultural library’. If that is indeed the case, it would be possible to think of interculturalism as a particular multicultural theory that has stressed some specific elements that other streams within the multicultural paradigm have somewhat neglected. According to Meer, Modood and Zapata-Barrero (2016), the (potential) points of tension between multiculturalism and interculturalism are fourfold. So, if interculturalism has an added value or is to be treated as something new, it has to do with these four points:

1. The status of dialogue, contact, and interpersonal relations. The intercultural paradigm would stimulate the interaction among people with different backgrounds, this can be done in the public realm, streets, library, schools, etc. (e.g. Zapata-Barrero, 2016, 2017)
2. The position of historical majority cultural forms. Interculturalism (at least, the version of Bouchard, 2011) would address multiculturalism’s alleged asymmetry in focusing only on the ethnocultural ‘minority’.
3. The normative significance of recognizing groups in addition to individual citizens. Some interculturalists seem to be hostile to the recognition of minority groups and argue that interculturalism prioritises individual over group rights (e.g. Cantle, 2016; Zapata-Barrero, 2016)
4. The status of minority religious communities and organizations. Interculturalists would not include religious groups within their framework as they put their trust in the approaches of toleration within existing secularist arrangements.

According to some authors – like Zapata-Barrero (2017) in this Special Issue – the intercultural ideas are indeed so fundamentally different that they can no longer be subsumed under the multicultural flag. These authors thus align with the narrative that multiculturalism has failed and needs to be replaced by another type of philosophy and policy. Take this quote of Zapata-Barrero (2016, p.54): *“IC enters into this negative diagnosis of MC, offering a lifeline. The epicenter of the debate in Europe is that MC has neglected intergroup relations and interpersonal contact among people with different origins and cultures. IC positions itself in contrast to both MC and assimilationism, based on substantial insights on the view of ethnicity and collective identity, as self-ascribed, flexible, and dynamic, and emphasizing the need for contact among culturally defined enclaves (which foster neither mutual identification nor interaction).”* While interculturalism might have increasingly become the new key signifier that unites critiques against multiculturalism, some multicultural scholars – like Modood (2017) in this Special Issue – are not impressed and argue that the interculturalism frame does not bring much new to the fore. *“(…) we conclude that until interculturalism as a political discourse is able to offer an original perspective, one that can speak to a variety of concerns emanating from complex identities and matters of equality and diversity in a more persuasive manner than at present, it cannot, intellectually at least, eclipse multiculturalism”* (Meer & Modood, 2016, p. 48). Interculturalists, as it is argued, would simply misrepresent the multicultural argument to make it more vulnerable to attack.

While the multiculturalism-interculturalism dispute might be just one of the many academic debates, Kymlicka (2016) warned that one should not take the issue too lightly, as ‘the good interculturalism versus the bad multiculturalism discourse’ might eventually play into the hands of assimilationists and xenophobes who reject both multiculturalism and interculturalism. Also Taylor (2016) suggests that, when the dust has settled, the most important lesson to be retained is that interculturalists and multiculturalists are engaging in an intramural fight, as most authors who work and defend their ideas under the banner of ‘multiculturalism’ or ‘interculturalism’ seek integration without assimilation.

### European & Canadian interculturalism

In Europe, the idea of interculturalism as a ‘counter’ to multiculturalism has been reflected in the White Paper on Intercultural Dialogue published by the Council of Europe (2008). According to the Council, the intercultural approach would avoid the failed extremes of assimilationism and multiculturalism by acknowledging diversity while insisting on universal values. This document emphasized the promotion of contact in order to enhance inclusion of immigrants. The European Council (2011) has also launched the Intercultural Cities Programme in order to support cities in reviewing their policies through an intercultural lens and develop comprehensive intercultural strategies to help them manage diversity positively and realize the diversity advantage. Intercultural integration, as Guidikova (2014, p. 1) argues, implies ‘*a strategic reorientation of urban governance and policies to encourage adequate representation, positive intercultural mixing and interaction, and institutional capacity to deal with cultural conflict*.’ Wood and Landry (2008), in fact, were amongst the first to emphasize ‘the diversity advantage’. They were also among the first to propose the image of the ‘intercultural city’, as the city was believed to be/become the most appropriate place for intercultural policies to prevail. ‘Proximity’ became the central axis for intercultural policies. As such the question about how contact needs to be managed in the public sphere (schools, streets, festivals, parks, libraries, etc.) became pivotal. This emphasis on the micro level, where face-to-face relations can develop, was believed to be highly neglected by multiculturalism, which has concentrated too much on ensuring the cultural rights of ethnocultural groups. As such, the intercultural approach heavily relies on the social contact hypothesis as initiated by Allport (1954). At this point the work of Cantle (2001, 2008, 2012, 2016) became influential. According to Cantle, a cohesive community is one where (1) there is common vision and a sense of belonging for all communities; (2) the diversity of people’s different backgrounds and circumstances are appreciated and positively valued; (3) those from different backgrounds have similar life opportunities; and (4) strong and positive relationships are being developed between people from different backgrounds in the workplace, in schools and within neighbourhoods. The concept of ‘community cohesion’ was invoked by Cantle following some riots in a number of towns in England in the summer of 2001. His approach emphasized the facilitation of interpersonal contact and intercultural competences to break down prejudices, stereotypes and misconceptions of others, and to generate mutual understanding, reciprocal identification, societal trust, social mix and solidarity. His ideas were laid out in the ‘Cantle report’ (Cantle, 2001), which then inspired several intercultural policies widely embraced in the UK and other countries by (local) government, public service and voluntary agencies.

The interculturalist raison d’être, so to speak, is that interaction among people has been overlooked by the multicultural citizenship paradigm, which has mainly concentrated on ensuring the cultural rights of diverse groups. Interculturalists like Zapata-Barrero, (2016, 2017) hold a plea for mainstreaming, hence the need for policy approaches that speak to the entire diverse population. Not only he, but also Cantle (2016) and other scholars bring to the fore the idea that people can have more than one identity at the same time and that these are not necessarily in opposition to each other, as they represent different aspects of human relations. Cantle, for example, argues that while the focus of multiculturalism on inequalities was justified, it has failed to adapt to the rising superdiversity of our societies and the multifaceted aspects of difference and otherness. Relying on one type of identity does injustice to the other identities (see also Levrau, 2018).

While the European intercultural approach thus emphasized the importance of contact and dialogue in a changing societal context of superdiversity, the need for community-cohesion and common bonds, and the significance of the micro-level where people actually meet each other, the intercultural approach of Quebec, however, is considerable different. In Europe interculturalism focused on the relations among citizens and groups in civil society, rather than on the relation of the state and its cultural minorities, which might be considered as the predominant concern of multiculturalism. On this basis, interculturalism and multiculturalism could be considered as compatible and even complementary strategies of integration (see e.g. Levey, 2016; Levrau & Loobuyck, 2013b; Loobuyck, 2016). The Quebecois interculturalism, however, − which, to a certain extent bears some similarities with the aforementioned ‘multicultural liberal nationalism’ approach of Kymlicka – is defined in opposition to the federal Canadian multiculturalism. Quebec hereby seeks to protect its French language and culture against the tide of Anglophone Canada. Ghosh (2011, p. 7) has summarized it in the following way: *“While Canadian multiculturalism is built on the assumption of not point to a dominant culture, interculturalism in Quebec is based on the understanding of the predominance of francophone culture: to build and integrate other cultural communities into a common public culture based on the French language, while respecting diversity.”* An influential voice here is Bouchard (2011), who argues that it is necessary to develop a form of pluralism that acknowledges that the francophone majority is itself a precarious minority that needs protection in order to ensure its survival and development in North America and in a globalized World. Remarkably, and yet another illustration of the confusing use of terms, Cantle (2016) argues that the Bouchard plea for interculturalism is difficult to sustain. This is because Bouchard’s interculturalism would mirror the reified, static and defensive form of identity management which would be typical for European multiculturalism and which Cantle wants to avoid and overcome through his ‘European interculturalism approach’.

### Overview of the contributions

The question whether or not interculturalism provides a new paradigm that transcends multiculturalism is the central question in this Special Issue. If we look at how the debate has been held so far, we can see that both hard and soft claims have been proposed (see also, Levey, 2016). Scholars like Bouchard (2011) and Cantle (2012) play it hard and argue that interculturalism is fundamentally different from multiculturalism. Others have argued that the distinctiveness is rather a matter of emphasis. Parekh (2016), for example, defends a non-combative approach by invoking the terms ‘multiculturally sensitive interculturalism’ and ‘interculturally attuned multiculturalism’, referring to the fact that both multiculturalism and interculturalism can learn from each other. Also Levrau and Loobuyck (2013b) and Loobuyck (2016) have emphasized and illustrated how both paradigms may be different but, more importantly, are complementary. Zapata-Barrero (2016, 2017) agrees to a certain extent that multiculturalism and interculturalism are complementary, but he focuses on the dividing lines and defends an encompassing theory that founds the ‘intercultural turn’. Meer and Modood (2016), in turn, have argued that interculturalists are pretty much barking up the wrong tree, as much of what interculturalism has brought to the fore can already be found in the multiculturalism paradigm.

In this Special Issue, several scholars shed their light on the debate and clarify their position. Tariq Modood and Ricard Zapata-Barrero, two of the leading proponents of this debate, each provide a separate and independent key text which is critically assessed by several authors. These scholars not only have specific academic expertise (political theory, political philosophy and sociology), but also work in countries that have different experiences with the multiculturalism-interculturalism debate (i.e. Belgium, Canada, France, Germany and the Netherlands). The issue starts with the contribution of *Zapata-Barrero* who defends the ‘intercultural turn’. In his view multiculturalism can be blamed for having neglected the importance of ‘contact’, ‘cohesion’, and what people have in common. As such, the intercultural approach is much more suited for the superdiverse societies of today and the future. *Modood’s* main idea is that interculturalists engaged with too much of a caricature of multiculturalism. At its best, interculturalism is to be understood as a version of multiculturalism, rather than an alternative. While most issues are already to be found within the multiculturalism paradigm, Modood nevertheless makes an exception for majority elements that he (and other multiculturalists) have to a certain extent neglected. In his commentary, *Christian Joppke* argues that there are several problems with interculturalism, such as the vacuity of the local as its preferred site of intervention, and its rushed embracing of ‘diversity’ that is also a central plank of neoliberal ideology. Also *Tamar de Waal* takes up a rather critical stance towards the intercultural position. She criticizes the widely spread image of a promising interculturalism that would come after a disappointing multiculturalism. The more multiculturalism is criticized, she argues, the more it will be difficult to implement pro-diversity strategies (including interculturalism). In their contribution, *François Boucher and Jocelyn Maclure*, disentangle both paradigms into separate elements in order to brew a ‘multicultural-intercultural position’ that is optimally adapted to the current superdiverse societies. In this commentary also *Stijn Oosterlynck* does not choose sides, but seeks to integrate elements of both paradigms. Oosterlynck engages in what he calls an ‘on-the-ground empirical analysis’ of how new forms of solidarity in diversity emerge. *François Levrau* provides yet another way to bridge both paradigms as he makes a plea for what he coins an ‘interpersonal ethos’. He contends that multiculturalism and interculturalism can only be successful in their shared ambition of the creation of a well-ordered and stable society in which the integration of all citizens is realized, on an equal basis, if these citizens act on the basis of a specific ethos that guides their many daily choices, interactions and behaviours. In her commentary, *Riva Kastoryano* highlights the emergence of transnationalism and deals with the extent to which this challenges the normative theories of multiculturalism and interculturalism. In a rejoinder, both Tariq Modood and Ricard Zapata-Barrero engage with the ideas that have been put forward by the commenting authors.

## Footnotes

1 This is why we will also mainly discuss multiculturalism in the context of immigration.

2 To mention just one categorization, Levey (2016) proposes the distinction between liberal and radical multiculturalism, which is about the extent to which the accommodation of cultural difference is limited by respects for liberal values. Depending on the purpose, Levey continues, one might further differentiate liberal (Kymlicka, 1995), democratic (Young, 1990), political (Modood, 2007) and pluralist (Crowder, 2002) models of multiculturalism, each emphasizing different fundamental values or ways of proceeding.

3 For a comprehensive country-by-country illustration and evaluation of multiculturalism policies related to immigrant minorities, see Tolley, 2011.

## References

[CR1] Allport G (1954). The nature of prejudice.

[CR2] Banting K, Kymlicka W (2006). Multiculturalism and the welfare state. Recognition and redistribution in contemporary democracies.

[CR3] Barry B (2001). Culture and equality. An egalitarian critique of multiculturalism.

[CR4] Bouchard G (2011). What is interculturalism?. McGill Law Journal.

[CR5] Cantle T (2001). Community cohesion: A report of the independent review team.

[CR6] Cantle T (2008). Community cohesion. A new era of cohesion and diversity.

[CR7] Cantle T (2012). Interculturalism: The new era of cohesian and diversity.

[CR8] Cantle T, Meer N, Modood T, Zapata-Barrero R (2016). The case for interculturalism, plural identities and cohesion. Multiculturalism and interculturalism. Debating the dividing lines.

[CR9] Council of Europe (2008). Living together as equals in dignity. White paper on intercultural dialogue.

[CR10] Council of Europe (2011). *The Intercultural Cities Programme (ICC)*. Retrieved from https://www.coe.int/en/web/interculturalcities/home.

[CR11] Crowder, G. (2002). *Liberalism and value pluralism*. London, New York: Continuum.

[CR12] Gesthuizen M, Van der Meer T, Scheepers P (2009). Ethnic diversity and social capital in Europe: Tests of Putnam’s thesis in European countries. Scandinavian Political Studies.

[CR13] Ghosh, R. (2011). The liberating potential of multiculturalism in Canada. Ideals and realities [Special Edition, spring]. *Canadian issues*, 3-8.

[CR14] Goodhart D (2006). Progressive nationalism: Citizenship and the left.

[CR15] Guidikova, I. (2014). *Cultural diversity and cities. The intercultural integration approach*. EUI: RSCAS policy paper 2014/02.

[CR16] Joppke C (2004). The retreat of multiculturalism in the liberal state: Theory and policy. British Journal of Sociology.

[CR17] Joppke C (2009). Veil: Miror or identity.

[CR18] Joppke C (2017). Is multiculturalism dead? Crisis and persistence in the constitutional state.

[CR19] Koopmans R (2010). Trade-offs between equality and difference: Immigrant integration, multiculturalism and the welfare state in cross-national perspective. Journal of Ethnic and Migration Studies.

[CR20] Kukathas C (2003). The liberal archipelago. A theory of diversity and freedom.

[CR21] Kymlicka W (1989). Liberalism, community and culture.

[CR22] Kymlicka W (1995). Multicultural citizenship. A liberal theory of minority rights.

[CR23] Kymlicka W (2001). Politics in the vernacular. Nationalism, multiculturalism, and citizenship.

[CR24] Kymlicka W (2002). Contemporary political philosophy.

[CR25] Kymlicka W (2007). Multicultural odysseys. Navigating the new international politics of diversity.

[CR26] Kymlicka, W. (2012). *Multiculturalism: Success, failure, and the future*. Washington, DC: Migration Policy Institute.

[CR27] Kymlicka W, Meer N, Modood T, Zapata-Barrero R (2016). Defending diversity in an era of populism: Multiculturalism and interculturalism compared. Multiculturalism and interculturalism. Debating the dividing lines.

[CR28] Levey GB (2001). The political theories of Australian multiculturalism. University of New South Wales Law Journal.

[CR29] Levey GB, Meer N, Modood T, Zapata-Barrero R (2016). Diversity, duality and time. Multiculturalism and interculturalism. Debating the dividing lines.

[CR30] Levrau, F. (2018). Pluriform accommodation: justice beyond multiculturalism and freedom of religion. *Res philosophica, 95*(1), 85–112.

[CR31] Levrau F, Loobuyck P (2013). Is multiculturalism bad for social cohesion and redistribution?. The Political Quarterly.

[CR32] Levrau F, Loobuyck P (2013). Should interculturalism replace multiculturalism? A plea for complementariness. Ethical Perspectives.

[CR33] Loobuyck P, Meer N, Modood T, Zapata-Barrero R (2016). Towards an intercultural sense of belonging together: Reflections on the theoretical and political level. Multiculturalism and interculturalism. Debating the dividing lines.

[CR34] McGhee D (2008). The end of multiculturalism? Terrorism, integration and human rights.

[CR35] Meer N, Modood T, Meer N, Modood T, Zapata-Barrero R (2016). Interculturalism, multiculturalism and citizenship. Multiculturalism and interculturalism. Debating the dividing lines.

[CR36] Meer N, Modood T, Zapata-Barrero R, Meer N, Modood T, Zapata-Barrero R (2016). A plural century: Situating interculturalism and multiculturalism. Multiculturalism and interculturalism. Debating the dividing lines.

[CR37] Modood T (2007). Multiculturalism. A civic idea.

[CR38] Modood, T. (2017). Must interculturalists misrepresent multiculturalism? *Comparative Migration Studies, 5*. 10.1186/s40878-017-0058-y 10.1186/s40878-017-0058-y PMC5591349 28955653

[CR39] Modood T, Triandafyllidou A, Zapata-Barrero R (2006). Multiculturalism, muslims and citizenship: A European approach.

[CR40] Okin SM (1998). Feminism and multiculturalism: Some tensions. Ethics.

[CR41] Parekh B (2000). The future of multi-ethnic Britain: Report of the commission on the future of multi-ethnic Britain.

[CR42] Parekh B (2000). Rethinking multiculturalism. Cultural diversity and political theory.

[CR43] Parekh, B. (2008). *European liberalism and ‘the Muslim question’ *(ISIM paper 9). Leiden: Amsterdam University Press.

[CR44] Parekh B, Meer N, Modood T, Zapata-Barrero R (2016). Afterword: Multiculturalism and interculturalism. A critical dialogue. Multiculturalism and interculturalism. Debating the dividing lines.

[CR45] Patten A (2014). Equal recognition. The moral foundations of minority rights.

[CR46] Pearce N (2004). Diversity versus solidarity: A new progressive dilemma. Renewal: A Journal of Labour Politics.

[CR47] Phillips A (2007). Multiculturalism without culture.

[CR48] Putnam R (2007). E pluribus unum: Diversity and community in the twenty-first century. Scandinavian Political Studies.

[CR49] Rawls J (1971). A theory of justice.

[CR50] Raz J (1994). Multiculturalism: a liberal perspective. Dissent.

[CR51] Sandel M (1982). Liberalism and the limits of justice.

[CR52] Taylor C, Gutmann A (1992). The politics of recognition. Multiculturalism: Examining the politics of recognition.

[CR53] Taylor C, Meer N, Modood T, Zapata-Barrero R (2016). Foreword. Multiculturalism and interculturalism. Debating the dividing lines.

[CR54] Tolley E (2011). Multiculturalism policy index: Immigrant minority policies.

[CR55] Uslaner E (2012). Segregation and mistrust. Diversity, isolation, and social cohesion.

[CR56] Van der Meer T, Tolsma J (2014). Ethnic diversity and its effects on social cohesion. Annual Review of Sociology.

[CR57] Vertovec, S., & Wessendorf, S. (2010a). Introduction: Assessing the backlash against multiculturalism. In S. Vertovec, & S. Wessendorf (Eds.), *The multiculturalism backlash. European discourses, policies and practices* (pp. 1-31). New York: Routledge.

[CR58] Vertovec, S., & Wessendorf, S. (Eds.) (2010b). The multiculturalism backlash. European discourses, policies and practices*.* New York: Routledge.

[CR59] Walzer M (1983). Spheres of justice. A defense of pluralism and equality.

[CR60] Wood P, Landry C (2008). The intercultural city. Planning for diversity advantage.

[CR61] Young IM (1990). Justice and the politics of difference.

[CR62] Zapata-Barrero R, Meer N, Modood T, Zapata-Barrero R, R. (2016). Theorising intercultural citizenship. Multiculturalism and interculturalism. Debating the dividing lines.

[CR63] Zapata-Barrero, R. (2017). Interculturalism in the post-multicultural debate: A defence. *Comparative Migration Studies*, *5*. 10.1186/s40878-017-0058-y 10.1186/s40878-017-0057-z PMC5583279 28944166

